# Circadian Rhythms, Exercise, and Cardiovascular Health

**DOI:** 10.5334/jcr.164

**Published:** 2018-07-12

**Authors:** Isabella M. Hower, Sara A. Harper, Thomas W. Buford

**Affiliations:** 1 Department of Aging and Geriatric Research, University of Florida, Gainesville, FL, US; 2 Department of Medicine, University of Alabama at Birmingham, Birmingham, AL, US

**Keywords:** circadian rhythmicity, circadian clock, entrainment, exercise, cardiovascular disease

## Abstract

Human circadian rhythmicity is driven by a circadian clock comprised of two distinct components: the central clock, located in the suprachiasmatic nucleus (SCN) within the hypothalamus, and the peripheral clocks, located in almost all tissues and organ systems in the body. Entrainment, or alignment, of circadian rhythmicity is dependent upon time of day and can occur through environmental influences such as light cues and physical activity exerted on skeletal muscle. Entrainment of the circadian clock through exercise has been reported to improve health by reducing risk of conditions such as cardiovascular disease (CVD), but further research is still needed. The purpose of this review is to discuss the effects exercise has on the regulation of circadian rhythmicity, specifically with respect to CVD risk factors – including hormonal levels, sleep/wake cycles, blood pressure, and heart rate. Additionally, the impact of exercise-induced circadian entrainment is discussed relative to hormone regulation, nocturnal blood pressure dipping, post-exercise hypotension, and overall cardiovascular health.

## Introduction

Cardiovascular disease (CVD) is the leading cause of mortality in developed nations [1,2]. Although many risk factors for CVD are known, emerging evidence suggests that alterations in circadian rhythms, which are daily cycles that regulate physiological functions, may contribute to CVD risk. The circadian clock controls circadian rhythms [3]. Alterations in circadian rhythms can be observed in hormonal secretion, blood pressure, heart rate, sleep, and other physiological processes [3].

Circadian rhythms are driven by an internal circadian “clock” that is responsible for regulating daily functioning of all major organs [4,5,6,7,8,9,10]. These rhythms are critical for maintaining a variety of beneficial health effects in humans [11]. The circadian clock can be categorized into two distinct components: the central clock and the peripheral clocks [8]. The central clock, located in the suprachiasmatic nucleus (SCN) found within the hypothalamus, serves as the fundamental factor for creating and maintaining circadian rhythmicity in mammals [6,7,9]. Entrainment, or alignment, of circadian rhythms in humans occurs directly as a result of photic and non-photic environmental cues [4,8,12,13,14]. Photic cues refer to light stimulus, while nonphotic cues refer to environmental factors such as feeding times, sleep-wake cycles, hormonal levels, and activity levels [12,13,14]. Additionally, peripheral clocks – found in almost all tissue and organ systems in the body – serve distinct roles in regulating circadian rhythmicity with respect to each location [8,13]. While the peripheral and central clocks can adjust to stimuli independently [8,11], photic stimuli that mainly influence the central clock can influence resulting circadian rhythmicity within the peripheral clocks [8,13].

Among the tissues regulated by clock activity, skeletal muscle represents a major organ system that influences human development as well as aging and disease [11]. It is thus important that skeletal muscle be synchronized with other organ systems and the environment directly through non-photic cues as well as indirectly through photic cues [15]. Similarly, one notable non-photic cue for regulating the peripheral clock is exercise [16]. Indeed, when photic cues were implemented along with non-photic exercise cues, entrainment occurred at a faster rate than those with exercise restriction [17]. Increasing knowledge regarding the circadian clock in humans has indicated that exercise has significant effects in regulating circadian rhythms, at least partly through its effects on skeletal muscle [7]. It is in part through these interactions with skeletal muscle that exercise may regulate circadian factors that influence cardiovascular health. For instance, deregulated circadian rhythms in skeletal muscle are associated with reduced glucose tolerance, as well as increased rates of diabetes, CVD, and cancer [11,15].

Meanwhile, exercise is a well-established method of improving cardiovascular health. Yet, less is known regarding the physiological effects of exercise on both the systemic and tissue-specific peripheral clocks, as well as the overall impact of these circadian effects on cardiovascular health [18]. Thus, the aim of this review paper is to explore the benefits of exercise related to the regulation and resynchronization of circadian rhythmicity and impacts on cardiovascular health in humans.

## Mechanisms of the circadian clock

The systematization of physiological and behavioral functioning from day to day, as seen in all mammals, is made possible by the intricate 24-hour day/night cycle initiated by the circadian clock [19,20] and is exhibited in both the central and peripheral clocks [20]. External photic and nonphotic environmental cues, also called zeitgebers, including light stimuli, time of feeding, ambient temperature, and exercise have the ability to influence and synchronize circadian rhythmicity [21,22]. Typical circadian rhythmicity occurring over a 24-hour period can be altered by non-photic cues; understanding these non-photic cues could lead to treatment strategies for the entrainment of the circadian clock [12]. Strategies may include adjusting sleep schedules [16,18,22] – such as in the case of shift work – as well as altering feeding patterns [21,23]. For instance, when shift workers sleep through the day, hypertension is often observed due at least in part to misalignment of circadian rhythmicity [9,23,24].

The SCN, which contains the central clock, processes external environmental stimuli such as photic cues [13,21] and internal input from the CNS [16]. As a result, the central clock entrains the peripheral clocks through the regulation of various rhythmic cycles including hormonal secretion, body temperature, sleep/wake cycles, blood pressure, and heart rate [16]. The most influential zeitgeber, light [21,22,25], signals the synchronization of bodily processes in both the central and peripheral clocks when the SCN receives light stimuli from the retina [21,22]. From there, the SCN can regulate circadian rhythmicity through sending signals to the peripheral clocks [21]. It is the circadian clock that enables animals, including humans, to adapt and respond appropriately to environmental changes observable in a 24-hour cycle [10,26]. Examples of these cyclical adaptations include daytime elevations/nighttime declines in core temperature as well as physical and mental functioning in response to environmental stimulation and activity levels [10,27]. While the presence or absence of natural light (photic cue) in a given 24-hour period serves to naturally synchronize circadian rhythmicity in the SCN [19,28], external nonphotic factors such as diurnal feeding cues and exercise have also been shown to promote circadian synchronization in peripheral tissue [16,19,22,29,30] through impacting the expression of clock genes [12,13].

## Circadian rhythmicity and cardiovascular disease

Daily circadian synchronization of physiological functioning is critical for the functioning of peripheral organs, including the heart [31]. Circadian misalignment over long periods of time is associated with elevated blood pressure [9,32], reduce sleep quality [9], and increase overall cardiovascular risk [1,9,23,31,32]. Incidence of CVD-related adverse events increase in frequency with diurnal variation [2,3,31]. Indeed, common CVD events – including sudden cardiac arrest, stroke, and myocardial infarction – predominantly occur in the early hours of the morning in response to rising heart rate and blood pressure [2,33] as well as an early morning release of hormones such as cortisol [34].

### Blood pressure

Several studies have reported that the circadian clock influences important CVD risk factors such as heart rate and blood pressure [1,11,18]. Circadian misalignment, even when short-term, can increase 24-hour blood pressure and decrease parasympathetic activity [23]. If circadian misalignment becomes chronic, ongoing elevation of blood pressure poses a risk factor for the development of CVD [23,34]. During the regular day/night pattern that follows a circadian rhythmicity, blood pressure drops during nocturnal sleep compared to diurnal wakefulness, which contributes to decreased stress to the cardiovascular system [35,36,37]. The nocturnal decrease in blood pressure compared to diurnal blood pressure is called “dipping” [35,36,37]. Nocturnal blood pressure that decreases by 10% to 20% of diurnal blood pressure levels is indicative of the dipping response [36,37]. Non-dippers, or those who do not exhibit the dipping response, are defined as having nocturnal blood pressure that decreases by less than 10% compared to diurnal blood pressure [35,36,38,39].

Compared to dippers, non-dippers have increased cardiovascular risk, including complications such as CVD and higher mortality [35,36,37] as well as greater complications for individuals already diagnosed with hypertension [35,38]. In individuals 60 years and older, increased systolic blood pressure accompanied by decreased diastolic blood pressure leads to increased pulse pressure and decreased dipping response at night [40]. These circadian variations of blood pressure in the elderly population lead to an increased risk of developing CVD [41,42]. Consequently, ambulatory blood pressure monitoring (ABPM) that tracks changes in blood pressure both diurnally and nocturnally can improve prediction of cardiovascular morbidity and mortality [35,43,44].

### Hormones and sleep

The increase and decrease in blood pressure in a 24-hour period due to physical and mental activity levels is influenced by the release of hormones, particularly cortisol and melatonin [3]. The central clock within the SCN signals the release of melatonin from the pineal gland at night [45,46], which correlates with lowered blood pressure and heart rate [3]. During the day, the adrenal gland follows a diurnal rhythm through the release of cortisol, which raises blood pressure and heart rate [3]. Due to the morning peak in cortisol levels and gradual decrease throughout the day [47], early morning release of hormones such as cortisol could contribute to this increase in blood pressure and resulting cardiovascular events [34]. Similarly, melatonin release is highly responsive to circadian rhythms and thus displays diurnal variation [23]. Among the key functions of melatonin is the regulation of sleep [14,23,45]. Often, the underlying cause of sleep disorders can be traced back to circadian misalignment between internal physiological processes and the external environment [3]. Among the most prominent areas where this is observed is in the aging process. As biologic age increases, the deterioration of daily functioning leads to the disruption of the circadian clock and circadian rhythmicity [4,48]. Not coincidently, these age-related changes are also associated with altered hormonal levels [7]. Of the many rhythmic changes associated with age, disruption of the sleep-wake cycle is commonly reported as a result of altered circadian rhythmicity and altered hormonal levels [4,49,50]. The incidence of increased wakefulness at night and drowsiness during the day are symptoms of disrupted circadian rhythmicity in the elderly [4,48] often contributing to adverse outcomes, such as falls, resulting from drowsiness [48]. “This age-related arrhythmicity of the sleep/wake cycle indicates a decline in functionality of the SCN at the cellular level and resulting circadian rhythmicity [4], leading to lowered amplitude of circadian rhythms as aging progresses [4,48].” It is possible that this decreased amplitude increases the tendency for internal desynchronization of circadian rhythmicity within the central clock in the elderly population [4,48], possibly leading to altered hormonal regulation and arousal patterns [48]. One study estimated that as many as 40–70% of the elderly population are affected by chronic sleep disruptions as a result of circadian misalignment [50]. Disruptions in the sleep-wake cycle have been linked to decreased cognitive performance [49,50], cardiovascular conditions [1], and increased vulnerability to disease [50].

## Exercise

Increased stimulation within the SCN through exposure to bright light is one proposed method of re-regulating circadian rhythmicity [49,50]. Similarly, the use of physical exercise may also serve as a viable option for restoring dysregulated circadian rhythms [50], likely due at least in part to changes in skeletal muscle. Skeletal muscle is an integral component of the peripheral clock that is functionally influenced by the circadian clock primarily through photic light cues [6,7,16]. Central clock genes in skeletal muscle regulate tissue specific biological processes such as diurnally and nocturnally influenced levels of skeletal muscle activity [16]. Circadian misalignment has been shown to have an adverse effect on peripheral clock genes and result in skeletal muscle dysfunction [16]. When circadian rhythmicity is altered in skeletal muscle, reduced glucose tolerance and changes in muscle functioning and composition can have a significant correlation with conditions such as diabetes, cardiovascular disease, and cancer [11]. However, further studies are needed to better understand the mechanisms of the circadian clock concerning skeletal muscle functioning [6,16].

**Figure 1:**
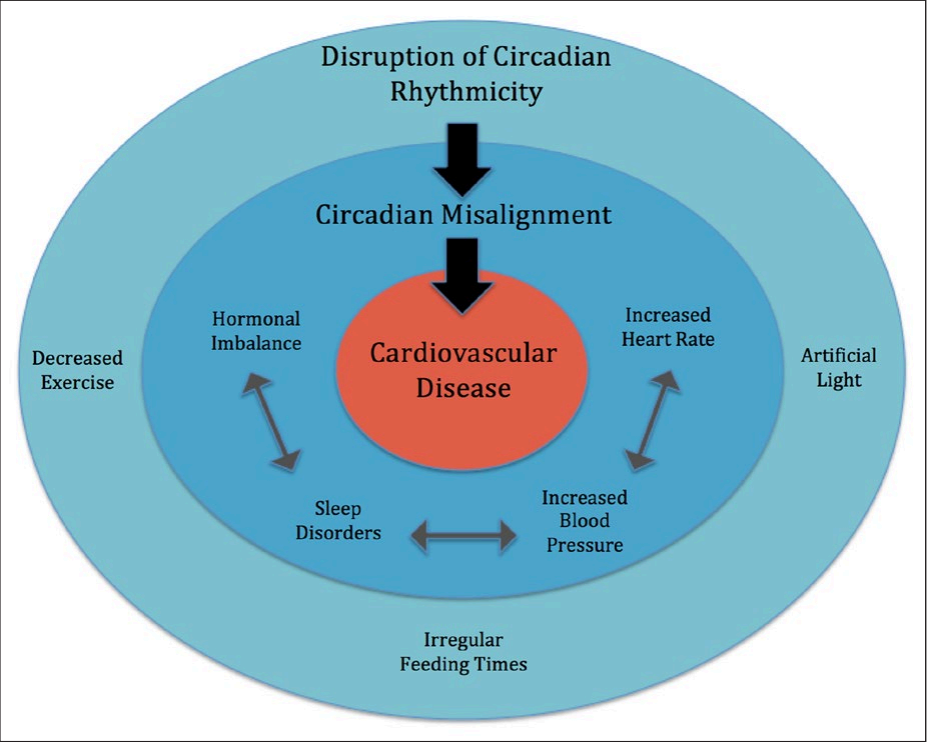
Conceptual figure outlining the potential impacts of physical exercise on circadian rhythms and downstream effects of these interactions on cardiovascular risk factors.

Increasing knowledge has made clear that exercise has a significant effect on the circadian clock [7,25], secondary exclusively to bright light [25]. However, though less recognized, several studies have demonstrated that the non-photic cue, exercise, has similar effects on entrainment of the circadian clock and the sleep/wake cycle to that of photic light stimulus [14,16]. Exercise has been widely shown to positively impact cardiovascular functioning, but the intricate link between exercise and synchronization of circadian rhythmicity has yet to be fully elucidated [18]. In addition to regulating cardiovascular functioning, circadian rhythms and the implementation of exercise on skeletal muscle have also been shown to influence and regulate hormones, blood pressure, and heart rate [24] (Figure 1).

Hormonal regulation and resynchronization of the circadian clock as a result of aerobic exercise have each been linked to improved sleep quality, lower heart rate, and lower blood pressure [51]. Regular exercise bouts led to drastic changes in hormonal regulation as a result of stimulating the neuroendocrine system [52]. For instance, cortisol levels have been shown to decrease at night by a larger margin with than without exercise [52,53,54]. Similarly, exercise promotes the production and release of melatonin and commonly results in improved sleep quality [45]. While the exact duration and intensity of exercise required to alter circadian rhythmicity has not been determined, one study in mice found that low-intensity endurance exercise sustained over the course of four weeks was adequate enough to entrain the circadian clock and alter circadian rhythmicity [28].

Comparably, moderate aerobic exercise helps reduce cardiovascular risk factors by lowering blood pressure at least in part due to adaptation to post exercise hypotension (PEH) [36,55,56]. While the influence of PEH and sleep structure has not been fully understood, some studies have found that fluctuation in PEH was greater after aerobic exercise in the afternoon as opposed to the morning [55,56]. However, it should be noted that PEH following morning exercise may be hidden by the circadian increase in blood pressure that occurs in the morning [55]. Alternative studies have concluded that PEH that occurs after morning aerobic exercise conducted at 7:00 am versus 1:00 pm may decrease nocturnal blood pressure and elicit lower PEH in general [55,57]. One study found that, while aerobic exercise performed both in the morning and the afternoon/evening contributed to PEH, when circadian influences of morning blood pressure were considered, PEH was greater following morning exercise rather than evening exercise [55].

Individual sleep patterns can also impact the optimal timing of exercise. Sleep duration and onset time depend on the chronotype, the natural propensity to sleep at a particular time, of each individual [5,6,10]. Indeed, studies have suggested that optimal diurnal exercise times vary based on individual chronotype [6,58,59]. Preliminary findings suggest that individuals, depending on generalized circadian rhythmicity patterns and sleep-wake cycle, can be categorized into three distinct groups: early circadian chronotype, intermediate circadian chronotype, and late circadian chronotype [6,60]. Regarding time of exercise, optimal skeletal muscle performance varies widely based on individual chronotype [6,60]. For instance, individuals with an early chronotype may want to exercise in the morning while individuals with a late chronotype might exercise in the evening to achieve optimal skeletal muscle performance [6,60]. Diurnal aerobic exercise, regardless of time and individual chronotype, also appears to support a healthy nocturnal dipping response in blood pressure [36,37,39]. However, optimal timing still appears to be an open question. One study found that, for non-dippers, aerobic exercise conducted in the evening had the greatest decrease in nocturnal blood pressure as opposed to aerobic exercise conducted in the morning [39]. However, another study has indicated the opposite, with exercise conducted at 7:00 am showing the most beneficial results for a reduction in nocturnal blood pressure as opposed to aerobic exercise conducted at 1:00 pm or 7:00 pm [57]. Based on the results of this study, it was concluded that exercise performed early in the morning enhanced sleep quality that in turn elicits a greater dipping response in nocturnal blood pressure [57]. Diurnal exercise is also recommended by the National Sleep Foundation as a nonpharmacological method for improving sleep quality [36]. Indeed, sleep quality and duration of sleep are higher in individuals who regularly engage in physical activity than those who do not [57]. This is a critical aspect of exercise, as a strong positive correlation exists between insufficient sleep quality and comorbid conditions – with less than 6–7 hours of quality sleep/night indicative of increased mortality, stroke, hypertension, and obesity [57].

## Conclusion

Misalignment of circadian rhythms has been indicated as a causal factor for altered heart rate, blood pressure, and hormonal levels [3,24]. With prolonged circadian misalignment as a result of these alterations, adverse cardiovascular conditions such as CVD can develop [1,9,23,31,32]. However, synchronization of circadian rhythmicity can reverse these adverse cardiovascular events and contribute to entrainment of the circadian clock [4,8].

Many studies have evaluated the separate effects of exercise, hormones, cardiovascular conditions, blood pressure, and other physiological aspects influenced by circadian rhythmicity and the circadian clock. However, to date, no research has been done to examine the holistic influence of exercise on circadian rhythmicity. While the mechanisms behind circadian rhythmicity on skeletal muscle functioning have not been fully elucidated, it is apparent that time of exercise can influence and elicit a change in circadian rhythmicity throughout the body by entrainment of the circadian clock [6,16,19,28]. Several studies have concluded that non-photic moderate diurnal aerobic exercise can promote circadian alignment similar to that seen through light stimuli [22,61]. Though it is well known that exercise improves cardiovascular health, the combination of timing, duration, and type of physical exercise regarding the efficacy of circadian alignment needs further examination [62]. Early evidence indicates that exercise can promote the synchronization of circadian rhythmicity and ultimately reduce the risk of chronic conditions, such as cardiovascular disease, through entrainment of the circadian clock to follow a regular 24-hour day/night cycle [20,22,62]. However, the extent to which these circadian improvements contribute to cardiovascular changes relative to other physiologic mechanisms remains unknown.

In conclusion, while it is widely accepted that circadian rhythmicity is positively influenced by exercise, further studies in this area are needed. The timing, duration, and type of exercise are particularly important in measuring the resulting benefits associated with circadian rhythmicity and should be addressed in future research. Taking this into consideration, determining the optimal exercise routine for maintaining circadian alignment and the resulting hormonal implications on heart rate and blood pressure could further aid in reducing cardiovascular disease risk.
